# Treating depression in Muslim communities: Meta-analysis of three trials of culturally adapted behavioural activation for Muslims (BA-M)

**DOI:** 10.1017/gmh.2026.10245

**Published:** 2026-06-11

**Authors:** Ghazala Mir, Robert West, Evrim Anik, Shaista Meer, Saima Dawood, Nazish Habib

**Affiliations:** 1 Leeds Institute of Health Sciences, University of Leeds, UK; 2 Leeds Institute of Cardiovascular and Metabolic Medicine, University of Leeds, UK; 3 Centre for Clinical Psychology, University of the Punjab, Pakistan; 4 Punjab Institute of Mental Health, Pakistan

**Keywords:** cultural sensitivity, depression, health inequalities, religion, decolonisation

## Abstract

Faith-sensitive depression therapies address a core aspect of cultural values but are not specifically promoted within global mental health policy. This study evaluated a culturally adapted Behavioural Activation intervention for Muslim patients (BA-M) compared with standard treatments across diverse settings. A patient-level meta-analysis combined data from two randomised controlled trials and one feasibility trial, comparing BA-M with cognitive behavioural therapy (CBT) and, in the UK, social interventions. The primary outcome was post-treatment depression severity (PHQ-9) and retention was analysed using Poisson regression alongside qualitative evaluation. A total of 266 participants were recruited (Turkey: 22; Pakistan: 103; UK: 141), with no baseline differences in depression severity between groups. BA-M produced significantly greater reductions in PHQ-9 scores, with an adjusted mean difference of −2.72 (95% CI: −3.93 to −1.51). Engagement was also higher in BA-M (IRR: 1.30; 95% CI: 1.18–1.44). Qualitative findings (n = 66) indicated strong support among patients, therapists and service managers, highlighting increased patient motivation and engagement in both Muslim majority and minority contexts. Participants emphasised the importance of religious identity in recovery from depression. The faith-adapted therapy BA-M demonstrates superior effectiveness to CBT and social interventions, supporting the integration of faith-sensitive approaches into global mental health practice.

## Impact statement

This research highlights the importance of religious identity to Muslim mental health. We demonstrate that faith-sensitive depression treatment is more effective than standard treatments in this population, which constitutes the second-largest faith group globally. Our three trials introduced inclusive policy and practice approaches within involved mental health services and staff trained in BA-M continue to use the intervention. BA-M training has also been delivered to 10 NHS therapy teams in the UK and to psychology practitioners in the UK, Pakistan, Turkey, Indonesia and the UAE. University modules have been developed to teach future psychologists in the UK, Pakistan and Indonesia and further work is being planned in Qatar and the United Arab Emirates. The UK course for clinical psychologists will be delivered online and globally accessible to therapists wishing to deliver BA-M. BA-M was effectively delivered by non-specialists in the UK, providing evidence that it can increase capacity among healthcare staff to deliver depression treatment. The intervention also provides a model for faith-sensitive treatments in other religious groups, as the systematic review that informed our development of BA-M included faith-sensitive interventions for a wide range of religious populations. A PhD study is currently being conducted at the University of Leeds to adapt the self-help booklet for Hindus with depression in the UK. This contribution to evidence about effective treatments can potentially support the development of governmental policies globally for faith-sensitive interventions delivered at scale to reduce the burden of depression. Such a change in policy and practice should significantly improve the quality of life for those who consider religion a significant aspect of their identity and subsequently impact education, employment and trust in mental health services. Our results are likely to influence thinking about the most appropriate intervention for treating depression in Muslim and other faith groups and we make recommendations for future policy, practice and research development.

## Introduction

Depression affects 4.4% of the global population, with a far higher burden in low- and middle-income countries (World Health Organization, 2021). In England, one in six people lives with moderate to severe depressive symptoms (Office for National Statistics, 2022). Poor access to therapy and low rates of recovery compared to the general population have been found among Muslims in the UK, the second largest faith group, who make up around 3.4 million of the population (The Lantern Initiative CIC et al., 2021; Baker and Kirk-Wade, 2024; Choudhry and Mir, 2022). The prevalence of depression in Pakistan, the second largest Muslim majority country in the world (Hafeez, 2023), is estimated at around 10% of the population – approximately 20 million people (Nisar et al., 2019) – and higher estimates up to 36% have been found among women in the postnatal period (Husain et al., 2006) and in a large urban settlement in Karachi (Altaf et al., 2015). In Turkey, a prevalence rate of 4.4% (around 3.3 million people) has been reported for depression (World Health Organization, 2021).

Culturally adapted therapies are more effective for many populations than Western treatment models (Anik et al., 2021), and there is evidence that people from minority backgrounds living in Western contexts may identify with a cultural or religious background that is not acknowledged in such treatment (Islam et al., 2015; Wallace et al., 2016). Western secular norms have been found to influence dominant knowledge in the field of mental health treatment internationally and to routinely undermine religious identity (Hansdak and Paulraj, 2013). Individuals accessing psychotherapy in the global South may thus be faced with similar issues because psychologists are trained in and use Western therapy models (Altaf et al., 2015). There is growing recognition that equitable access and outcomes to mental health support involve decolonising global mental health systems, that is, removing systemic oppression and discrimination by challenging dominant norms that reflect Eurocentric systems of knowledge and power and perpetuate inequitable outcomes (Fay, 2018; Rivera-Segarra et al., 2022).

Behavioural activation (BA) has been successfully adapted for specific cultural communities such as Latino patients in America (Kanter et al., 2015) and can be delivered by non-specialist healthcare professionals (Ekers et al., 2011). BA is an evidence-based treatment with comparable efficacy to cognitive behavioural therapy (CBT) (Richards et al., 2016). Component techniques of BA include activity scheduling and monitoring, which are linked to an assessment of life goals explicitly linked to core values; skills training; contingency management and procedures targeting avoidance (Kanter et al., 2010).

Drawing on a previous adaptation of BA (Kanter et al., 2015), we developed a culturally adapted BA therapy for Muslims with depression (BA-M). The cultural adaptation is offered to patients who identify religion as a core value and incorporates: conceptualisations of depression that differ from Western psychiatric frameworks; attention to religious identity as a resource for health and as a trigger for discrimination; an understanding of cultural and religious influences on social relationships; and awareness of the availability of community resources within Muslim populations.

A pilot study on the intervention in the North of England provided evidence that BA-M was acceptable to both service users and therapists and feasible in practice within UK clinical settings (Mir et al., 2015). A subsequent feasibility study in Turkey explored the acceptability and feasibility of the intervention in that context (Anik, 2022). Two further RCTs in Pakistan (Dawood et al., 2023) and the UK (Mir et al., 2023) compared the efficacy of BA-M to the usual treatment of Cognitive Behavioural Therapy (TAU) in a Muslim majority and Muslim minority context. The UK trial also evaluated whether BA-M could be delivered by non-specialist voluntary sector staff trained in BA and BA-M.

In this paper, we present results across all three studies and a meta-analysis on the effectiveness of the BA-M therapy compared to TAU. Our results provide evidence of the effectiveness of BA-M in settings where Muslims are the majority population (Pakistan and Turkey) as well as where they constitute a minority group (UK).

## Methods

### Study designs

A cluster randomised controlled feasibility trial was conducted in Istanbul, Turkey (Anik, 2022). Therapists were recruited via social media advertisements promoted by two renowned CBT experts, a clinician and a university academic. Therapists then recruited participants via their usual caseload. A non-blinded randomised controlled trial was conducted in Lahore, Pakistan. Participants were recruited from, or self-referred to, outpatient departments of two hospitals and the Centre for Clinical Psychology at the University of Punjab (Dawood et al., 2023). In the UK, a non-blinded randomised controlled trial was conducted in Bradford. Recruitment was via NHS primary care mental health services and three voluntary sector mental health organisations (Mir et al., 2023).

Each trial was assessed for bias by at least three authors, using the Revised Cochrane Risk-of-Bias Tool for Randomised Trials (Higgins et al., 2019).

### Participants

All studies recruited adult participants who self-identified as Muslim, aged 16 years and over (over 17 in Turkey), residing within recruitment sites. Eligibility was determined through screening for depression using a combination of clinical interview and routinely used depression measures in each context: the Symptom Checklist-90 (Revised) Questionnaire (SCL-90-R) in Turkey; PHQ-9 in the UK, and both measures in Pakistan (Dawood et al., 2023; Mir et al., 2023). A co-morbid diagnosis of primary psychological disorder for which empirically supported treatments exist (e.g. PTSD or bipolar depression) was an exclusion criterion in all trials. Translated information sheets, consent forms and questionnaire measures were available in relevant languages. For participants with limited literacy skills, staff provided verbal explanations in a relevant language with support to provide informed consent and complete questionnaire measures. Written or verbal informed consent was recorded either at recruitment or before the first therapy session.

Demographic data on gender and age were collected for patients in each trial. In the UK, details of postcode were also collected, using routine categories collected for NHS primary care data. Participant details collected for the Pakistan study also included education, marital status, employment, socio-economic status and family system (nuclear/joint) (Dawood et al., 2023).

Therapist recruitment was through study sites involved in the Pakistan and UK trials and through social media advertisements in Turkey. Psychologists in all three settings had at least 1 year of training in psychological therapies and received 2 days of training on how to deliver BA-M.

In the UK trial, the intervention was also delivered by non-specialist staff with graduate-level education working in community mental health organisations (VSOs). All VSO staff in the intervention arm received a 5-day training course on BA delivered by a skilled academic who also assessed participant competency in BA prior to their receiving the BA-M training. The competency assessment involved a roleplay exercise used for a previously published trial of BA delivery by non-specialist providers (Ekers et al., 2011). Fidelity to the BA-M intervention was assessed through clinical supervision, provided to VSO providers for 2 hours each month by psychotherapists trained as clinical supervisors.

Monthly 1-hour peer support sessions were also offered to all BA-M therapists in the UK and Turkey trials to provide additional support and further monitor fidelity to the intervention. Discussions focused on practice experiences without naming specific clients and practitioner queries about implementing BA-M. Clinical supervisors attended the BA-M training and peer support sessions to further support fidelity during implementation. A statistical evaluation of the first 25 PHQ-9 measures collected at VSO sites was conducted in the UK to assess the safety of therapy delivery in VSO settings. Therapists in the control groups for the Pakistan and UK trials did not receive BA-M or BA training. In Turkey, control group therapists attended BA training and had bi-monthly meetings to support engagement with the study.

Patients and therapists in the BA-M arm were interviewed in all contexts to provide further insights into delivery and organisational issues; in the UK, therapy supervisors and organisation managers were also interviewed. Qualitative interviews explored the experience of the intervention in terms of: barriers and facilitators to access, acceptability, impact, reach, contextual and other influences on delivery/impact, mechanisms of impact and unanticipated effects. Separate consent was taken from clients in the BA-M arm at the recruitment stage to participate in the qualitative evaluation. Verbal informed consent was obtained if it was technically difficult to obtain written consent because of limited literacy. Interview data were translated, if necessary, transcribed and coded using NVivo software and analysed using framework analysis to identify key themes (Ritchie et al., 2003).

### Randomisation and masking

Randomisation in Turkey was at the therapist level. Nine therapists were randomly allocated by RW through a random number generator to either CBT as TAU or BA-M using a 1:2 ratio. In addition, to avoid contamination, three therapists who were unable to attend the BA-M training were directly allocated to TAU and two therapists who took part in translating BA-M materials to Turkish were allocated to the BA-M group.

In Pakistan, a blinded assessor screened patients for depression using the Urdu version of the PHQ-9 and a symptom checklist (SCL-90-R). Participants were recruited in pairs based on intention to treat and allocated to receive either the intervention or TAU using a random number generator (block randomisation). Randomisation to BA-M or TAU could not be concealed from patients or therapists.

In the UK trial, a blinded assessor screened patients for depression and patients were recruited based on intention to treat. Participants were then allocated to receive BA-M or TAU in blocks of 2.

In all three trials, blinding of therapists, patients and researchers was not possible for treatment or analysis.

### Procedures

Participants received either Behavioural Activation for Muslims therapy (BA-M) or TAU. BA-M consisted of 6–12 sessions of culturally adapted BA therapy, delivered by therapists trained in the intervention. A Values Assessment was used to identify the importance of religion and patients for whom this was an important value were offered the opportunity to use a self-help booklet to support their recovery. The booklet uses Islamic religious teachings to promote therapeutic goals and “positive religious coping” that is, an internalised understanding of religion that provides meaning, resilience, hope and self-esteem as a therapeutic mechanism for recovery from depression (Pargament, 1997; Pargament et al., 2000; Mir et al., 2015).

TAU was primarily CBT with some differences across the three sites. In Pakistan, TAU consisted of eight sessions of CBT and in Turkey, at least six sessions. In the UK, TAU was either six sessions of CBT in NHS primary care settings or an open-ended social intervention in community-based voluntary sector services. Adherence to the therapy manual was assessed through regular peer support meetings between researchers and therapists in the UK and Turkey and process evaluations in all contexts (Table 1).

**Table 1.** Intervention details by site Table 1. long description.

Sites and arms	Intervention dose (number of sessions)	Provider type	Supervision and fidelity assessment
*Turkey*			
BA-M	At least 6	Individual CBT-trained therapists	Clinical supervision Peer support meetings
TAU	At least 6		Clinical supervision
*Pakistan*			
BA-M	6–12	CBT-trained therapists in government-funded clinics	Clinical supervision
TAU	8		Clinical supervision
*UK*			
BA-M	NHS: 6; VSO: 6–12	NHS: CBT-trained therapists VSO: Staff trained in BA	Clinical supervision Peer support meetings Qualitative interviews
TAU	NHS: 6; VSO: open-ended	NHS: CBT-trained therapists VSO: mental healthcare staff	Clinical supervision

### Outcomes

Routinely used measures for screening and outcome were used in each context to align with usual practice; however, all sites used PHQ-9 as a common outcome measure. The primary outcome in both RCTs was the final PHQ-9 score, with scores recorded at every session in all trials. PHQ-9 score was a secondary outcome in the Turkey feasibility trial, with the primary outcome being recruitment and retention of therapists and patients (Anik, 2022).

In Pakistan, several secondary measures included activation, measured by the BADS-SF depression scale, and quality of life measured by SCL-90-R (Dawood et al., 2023). In the UK, secondary outcomes included anxiety measured by the GAD-7 and PHQ-9 scores at each session (Mir et al., 2023). Outcomes were collected in the UK by someone independent from the research and therapy delivery teams.

### Assessment of safety and adverse events

Routine reporting and supervision procedures were used at each participating site for assessment of safety and adverse events. Within the UK and Pakistan trials, this involved formal institutional supervision of therapists and reporting of adverse events. All therapists in these trials were regularly supervised by experienced clinical leads to discuss issues arising in therapy. Therapists could also contact supervisors to report concerns between supervision sessions. Patient feedback was continuously monitored by participating organisations, and a Project Steering Group was established for each study to address any adverse events or concerns about safety. In Turkey, therapists worked in the private sector and were asked to report concerns through contact with the lead researcher (EA) or at meetings with research team members.

Adverse events were defined as any unfavourable and unintended symptom or disease, including psychological, emotional or physical harm, occurring during the course of the trials. Incidence was monitored through clinical supervision sessions with therapists and, in the UK, early statistical evaluation of VSO depression measures to ensure that participants were not exposed to unexpected worsening of symptoms or harm from the intervention (Mir et al., 2023).

### Statistical analysis

The sample size for both RCTs was informed by previous research and consideration of potential drop-out. In Pakistan, 59 patients were required in each therapy arm to identify a minimal clinically important difference in PHQ-9 scores of 2 units with 80% power (Dawood et al., 2023). In the UK, with 5% (two-sided t-test) significance, a difference of 3 units could be detected with 90% power with 60 patients (Mir et al., 2023). The feasibility study in Turkey did not have a formal power calculation and was included to increase the total number of patients in the overall sample.

The observations from the two trials, together with the feasibility study, were combined and analysed together; thus, a one-stage patient-level meta-analysis was undertaken. Unlike a pooled regression, this involved patients from different populations rather than the same population (Stewart and Tierney, 2002; Riley et al., 2010). The primary outcome was taken as the final PHQ-9 measurement taken in the last attended therapy session. The main analysis was a regression of the final PHQ-9 score upon the baseline PHQ-9 score as well as therapy arm (TAU or BA-M) with a separate intercept for each study (Pakistan, UK and Turkey). That is, an ANalysis of COVAriance (ANCOVA) was performed to allow for different severity of depression in participants at baseline. No account for clustering within therapists was made, since such a clustering effect had been seen to be negligible in the Turkey and UK trials (Anik, 2022; Mir et al., 2023). The therapists’ details were not available from Pakistan. Observations where baseline PHQ-9 score data were missing were replaced with the first available PHQ-9 score. Observations where the final PHQ-9 score was missing were replaced with the score from the last available session.

A descriptive table was also produced to assess clinically meaningful change, which was defined as a 5-point or 20% reduction in PHQ-9 score (Löwe et al., 2004a; Kounali et al., 2022; National Collaborating Centre for Mental Health, 2024).

Since there were differences in how TAU was defined between organisations that delivered therapy within the UK study, a sensitivity analysis was conducted for this study, adjusting for the effect of organisation on therapy undertaken. Results showed that adjusting for the organisation had only a minimal impact on therapy effect (Supplementary Tables E and F show final PHQ-9 scores adjusted for baseline PHQ-9 and therapy group).

An interaction term between therapy type and study was added to test whether the effect of therapy differed between studies. There was little evidence of such an interaction, so this was dropped from the analysis. A Poisson regression was undertaken as further analysis, to assess retention rate by therapy arm and study, regressing the number of sessions attended upon the trial and the therapy type.

Data collection and analysis were monitored by a Steering Group of involved stakeholders (NHS and third sector service providers, health service commissioners and academics) for the UK trial and by the research teams for the Pakistan and Turkey studies. Both RCTs were registered with the UK’s Clinical Study Registry (see Data Availability statement).

## Results

Altogether 266 patients were randomised to receive either BA-M or usual treatment (TAU) across the three trials (Turkey: 22; Pakistan: 103; UK: 141) between 4 January 2019 and 31 March 2023. Findings from each study are reported below, along with a meta-analysis of the effectiveness of BA-M compared to TAU across all three trials.

### Turkey

Fourteen therapists were recruited between 4 January 2019 and 31 January 2020. Eight were randomised to offer BA-M and six to offer TAU. Of 23 patients recruited, 14 received BA-M and eight received TAU. One patient did not meet the eligibility criteria of identifying as Muslim and was removed from analysis (Anik, 2022).

### Pakistan

Recruitment took place from 1 March 2020 to 31 August 2021. Altogether 154 patients were assessed for eligibility, 114 participants were randomised (57 in each group), and the final sample consisted of 103 participants (53 BA-M; 50 TAU). Both groups reported participants who dropped out before follow-up measures could be collected (4 BA-M; 7 TAU) (Dawood et al., 2023).

### UK

193 patients were recruited from October 2021 to March 2023, 141 participants were included in analysis after screening: 81 randomised to BA-M and 60 to TAU (Mir et al., 2023).

**Figure 1.**
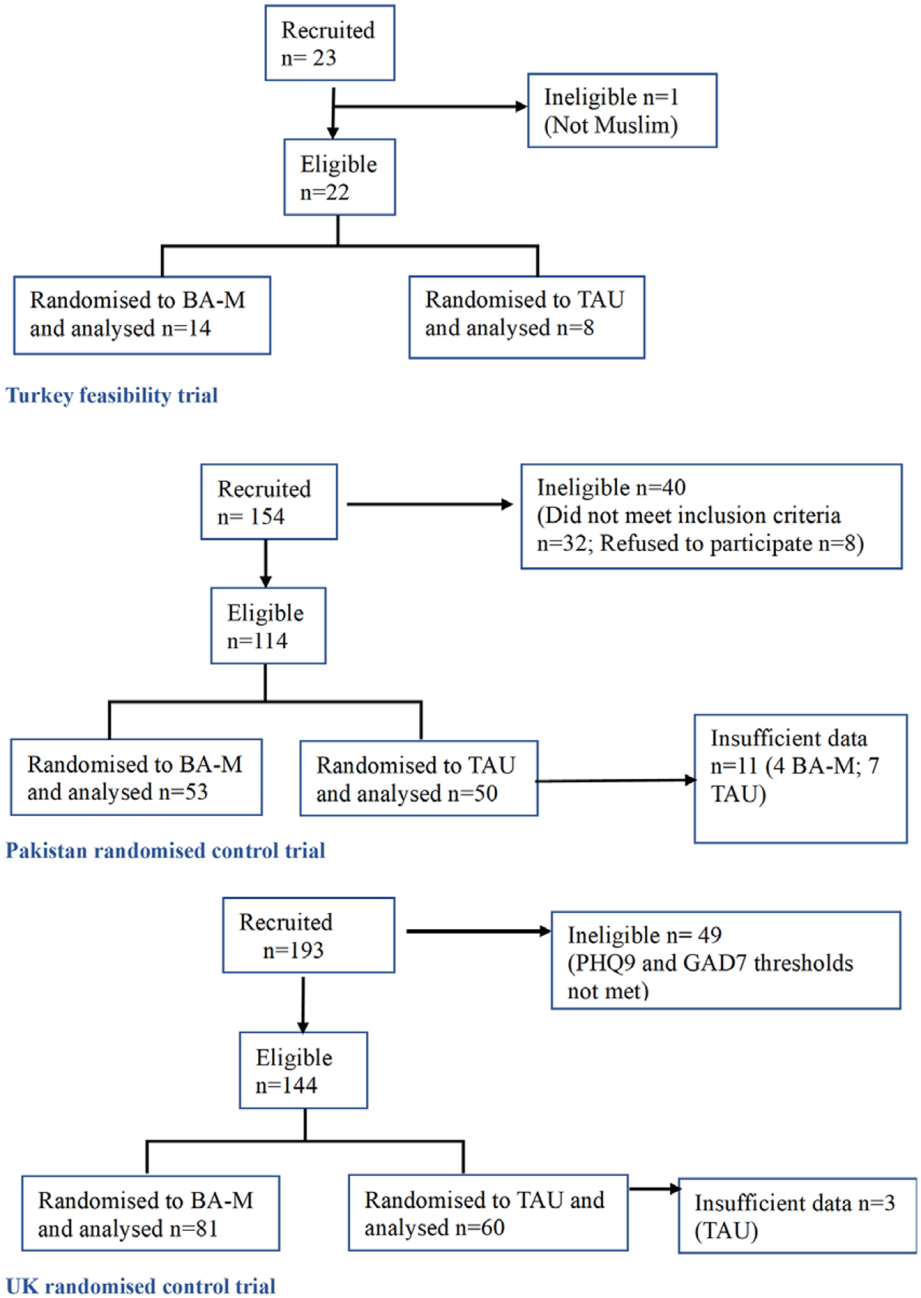
Consort flow diagrams for each trial. Figure 1. long description.

Participant flow diagrams below draw on the published study reports and Consolidated Standards of Reporting Trials statement (Hopewell et al., 2025) (Figure 1).

### Participant characteristics

Table 2 shows there were no significant differences between comparison groups in participants’ baseline characteristics across the three trials. More female participants were recruited in all three trials compared to male participants, but this was not significantly different between the BA-M and TAU groups. Patient age and baseline PHQ-9 scores in each trial were also not significantly different between the two groups.

**Table 2.** Baseline characteristics by therapy group Table 2. long description.

	BA-M	TAU	P test
*n*	148	118	
Study (*n*, %)			
Pakistan	53 (35.8)	50 (42.4)	0.48
Turkey	14 (9.5)	8 (6.8)	
UK	81 (54.7)	60 (50.8)	
Sex (*n*, %)			
Female	116 (78.4)	85 (77.3)	0.95
Male	32 (21•6)	25 (22.7)	
Age (mean (SD))	34.17 (10.42)	37.43 (12.71)	0.054
Baseline PHQ-9 score (mean (SD))	19.12 (4.54)	19.71 (3.87)	0.28

Ethnic group data were collected in the UK trial and showed that the sample was mostly from Asian or Asian British backgrounds. Ethnicity was not, however, significantly different between the two groups at baseline (*p* = 0.48). Baseline scores for an additional measure for anxiety (GAD-7) also found no significant difference between the two groups (*p* = 0.68). Deprivation status was not collected for all UK participants, but all of those for whom data were available (*n* = 33) were from deprived areas.

In Pakistan, a BA-specific measure (BAD-SF) did show a significant difference between the comparison groups at baseline (*p* = 0.001), with higher activity scores reported in the BA-M group, despite similar depression scores to TAU participants (Dawood et al., 2023).

### Findings for the primary outcome

**Figure 2.**
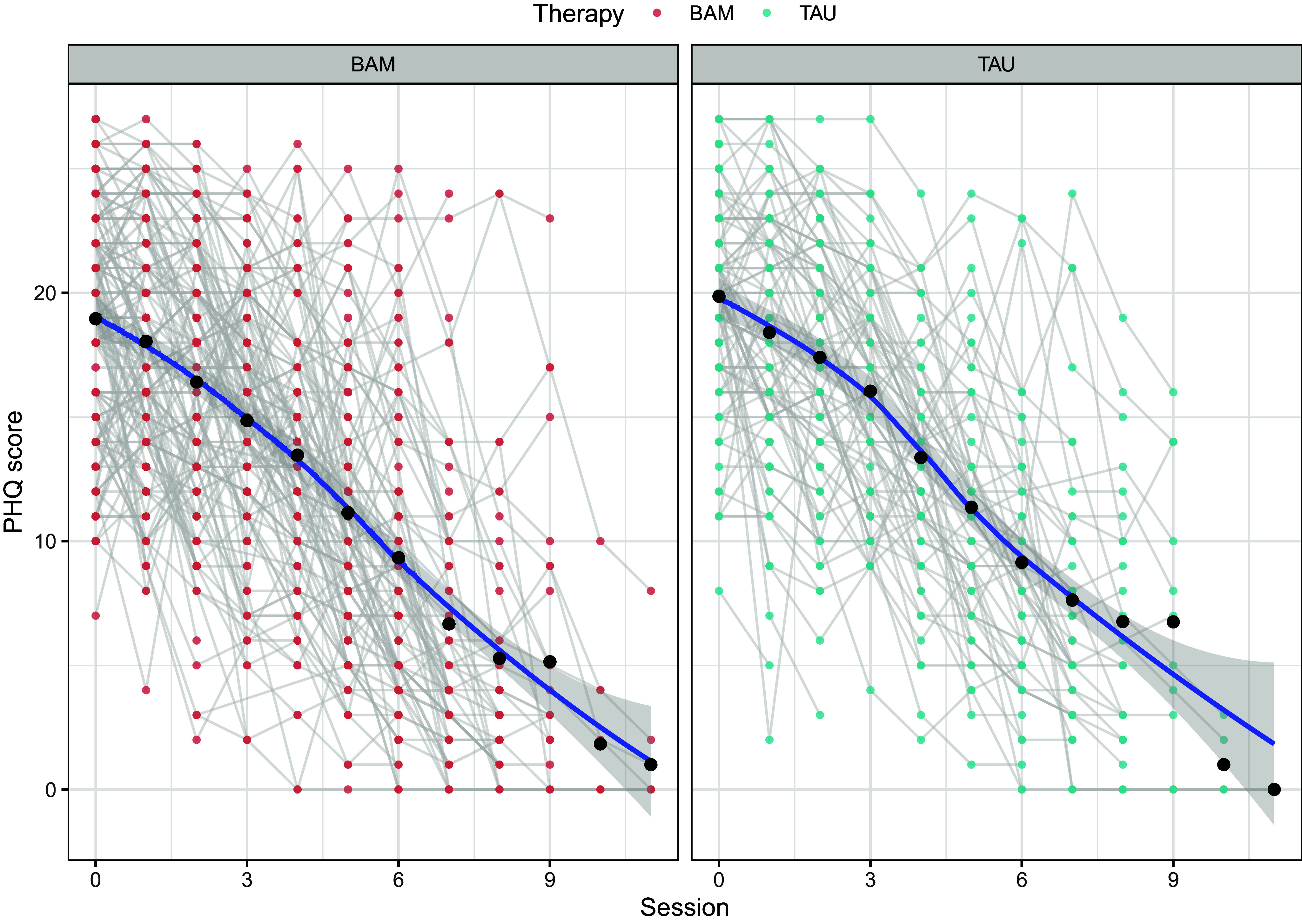
Trajectories of depression symptoms. Figure 2. long description.

Figure 2 presents differences in depression scores for each type of therapy. Across the three trials, the mean PHQ-9 score at the final session was 9.6 (SD = 7.16) for patients in the BA-M group compared to 11.95 (SD = 7.15) for the patients in the TAU group. BA-M group patients were also more likely to have more sessions (*M* = 6.63 (SD = 3.17) compared to TAU patients (*M* = 5.31 (SD = 4.45).

Regression results from the ANCOVA model in Table 3 show statistically and clinically significant differences in the final PHQ-9 reported by the study. Table 3 shows that after controlling baseline scores and study site, participants receiving BA-M therapy had significantly lower post-treatment PHQ-9 scores compared to the TAU – that is, a 2.72 points reduction (β: −2.72, 95% CI [−3.93,−1.51]).

**Table 3.** Coefficients from ANCOVA model used to regress final PHQ-9 scores on study, baseline PHQ-9 and the therapy group Table 3. long description.

Term	Estimate	95% CI	*p*-value
(Intercept)	−0.96	−4.03, 2.11	0.538
Turkey	3.63	1.23, 6.02	0.003
UK	10.40	9.13, 11.66	<0.001
Baseline PHQ-9	0.38	0.24, 0.52	<0.001
BA-M Therapy	−2.72	−3.93, −1.51	<0.001

Retention of patients was better with BA-M than with TAU, with an average of 6.6 sessions under BA-M and 5.3 for TAU. The Poisson regression results in Table 4 show statistically significant differences. Participants receiving BA-M had a 30% higher session attendance rate compared with TAU (IRR:1.30, 95% CI [1.18, 1.44]).

**Table 4.** Incidence rate ratios (IRR) from Poisson regression predicting the number of sessions attended by the study and therapy group Table 4. long description.

Term	IRR	95% CI	*p*-value
(Intercept)	7.59	6.95, 8.28	<0.001
Turkey	0.81	0.69, 0.96	0.015
UK	0.43	0.39, 0.48	<0.001
BA-M Therapy	1.30	1.18, 1.44	<0.001

Secondary outcomes varied across the Pakistan and UK studies and also showed significant differences. In the UK trial, anxiety, as measured by GAD-7 scores, was significantly lower (*p* = 0.001) for BA-M participants (*M* = 12.81) compared to TAU (*M* = 15.21) (Mir et al., 2023). In the Pakistan trial, “activation,” measured using the BA-specific BADS-SF scale, showed mean scores post-therapy were significantly different between the two groups with BA-M at 34.34 vs. TAU at 27.90 (*p* < 0.001) (Dawood et al., 2023).

The Pakistan trial also reported that patients were retained at a better rate in the BA-M group, compared to TAU. Patients who received BA-M were found to attend a mean of 9.0 sessions compared to 8.25 sessions in the TAU group (*p* = 0.013) (Dawood et al., 2023). In the UK, the mean number of sessions was much lower but still far better for BA-M (*n* = 3.83) than TAU (*n* = 1.5) (Mir et al., 2023). More than half of clients in the UK (51.7%) attended only one session of TAU compared to 13.6% for BA-M (Supplementary Table A).

Of the total 266 clients, 173 had a reduction of five or more points on PHQ-9 and 60.1% of these received BA-M. A similar percentage of the 182 clients who had > = 20% decrease in their PHQ-9 scores were treated by BA-M (59.9%; Supplementary Tables B and C).

Although heterogeneity was observed between studies, driven primarily by differences in baseline PHQ-9 scores, this variability was explicitly accounted for in the analysis. Despite these differences, all studies favoured the BA-M intervention, suggesting that the observed heterogeneity enhances rather than undermines the evidence for the consistent effectiveness of BA-M across populations with varying baseline symptom severity. A sensitivity analysis excluding the Turkey study, because of its limited power, indicated minimal effect on the results and did not affect the results of our meta-analysis (Supplementary Table D).

### Risk of bias assessment

The three included trials demonstrated some concerns about formal risk of bias assessment, consistent with real-world trial designs. These are primarily related to a lack of blinding for outcome assessors, which has been associated with an exaggerated effect estimate report (Juul et al., 2021). A self-reported measure for the primary outcome was also used, in line with common practice for the type of intervention. Despite these unavoidable limitations, the trials adhered closely to best practice standards and were free from high-risk sources of bias.

### Process evaluation

Qualitative data on the views and experiences of BA-M were collected from participants across the three sites. In Turkey (*n* = 18), eight BA-M clients, seven BA-M and three CBT therapists were interviewed. In Pakistan (*n* = 14), 12 BA-M clients and two BA-M therapists were interviewed; in the UK (*n* = 34), interviews in the treatment arm were conducted for 18 service users (15 who completed therapy; 3 who dropped out) and 9 BA-M therapists (4 NHS, 5 VSO). In addition, seven managers or supervisors (3 NHS, 4 VSO) were interviewed.

Data on intervention fidelity, gathered through formal supervision, peer support meetings and/or qualitative interviews (see Table 1), indicated that most therapists understood the essential elements of BA-M and gained confidence in delivery as they developed practice experience. Support to answer questions and discuss cases helped address misconceptions and the sharing of practical experiences in delivery (Anik, 2022; Mir et al., 2023).

Qualitative data helped explain our quantitative findings on the greater engagement of patients with BA-M than TAU (Table 5). For many patients, religion was a central feature of their identity and support to engage with religious teachings to develop positive religious coping during therapy was a key factor in their improved engagement. This approach helped them reconnect with religion as a resource for health, providing affirmation of their values, motivation, meaning, comfort, resilience and hope of recovery. Service users confirmed they needed support to be able to draw on their beliefs in this way and some felt BA-M was more “client-centred” and “authentic” than their previous experiences of mental health support.

**Table 5.** Qualitative findings Table 5. long description.

***Significance of religion in therapy***	“The whole therapy was to do with what was important to me as well, and religion was a part of it. I said that that is very important to me because I’ve got a strong faith, and I want to keep that. […] I didn’t want to lose hope and that was one of the things, I said religion was important to me.” (UK: AL, VSO Service User, female, completed) “religion is absolutely core.” (UK: KT, female, VSO service user, completed) “I started my studies again and returned to religion I started praying again. I started my studies with my own heart and in a good way. I felt this change.” (Pakistan, MM, Service User, female, completed) “Previously I used to think to myself ‘If I perform my prayers, if I become closer to Allah, my psychology would improve, I would have an objective in life.’ But, well, unfortunately we cannot always manage to do what we think we can.” (Turkey: FG, Service User, female, completed) “when I looked closely at the Booklet, I said to myself ‘These things are not outlandish things at all, these are things which I have always known about, but they are things which I have not incorporated into my life. In fact,’ I said to myself, ‘it is because I have not incorporated these things into my life that I am seeing a psychologist today.” (Turkey, ML, Service User, female, completed)
***Legitimacy of religious identity***	“there’s absolutely a need for it and I think it can be for certain clients an absolute priority and can make all the difference when somebody is acknowledging. […] if cultural and religion come up there high, then you want to be making sure that you are bringing that into the session. (UK: OI, NHS manager) “maybe in the past […] I’ve held away from using religion and culture and I was given this flexibility to use religion and culture and talk about it openly.” (UK: TH, female, VSO, therapist)
***Religious coping***	“we are Muslims, so there should be ample material on Islam, and we should also be taught from a religious perspective.” (Pakistan: MK, service user, male, completed) “it was great, as we are Muslim and we should understand things with respect to our religion” (Pakistan, AN, Service, User, female, completed) “It was great that the subject [religion] was brought up,” (Turkey: KB, service user, female, completed) “it kind of felt good in the sense of, well I’m having to put in this effort, […] It tallies with the verse in the Quran that you know, you’ve got to change your own situation you know, […] and that Allah will help you, so it kind of tallied with it. […] tie the camel first, yeah, and then just have your trust in Allah.” (UK: KT, female, VSO service user, completed) “What I found was a lot of the women were coming across as like you’ve been punished […]. According to some of these women you are supposed to be angels all the time and it’s like that’s not possible because you are human and that’s how you’ve been created and that you do sin.” (UK: BI, female, VSO therapist) In the area of punishment or past sins, then environmental factors can sometimes be mixed up with religion. […] [S/he feels] as if s/he has been punished. Again, in the structuring section we resolve this with Socratic dialogue: […]” (Turkey: RM, BA-M Therapist)
***Valuing client beliefs***	“there is automatically an extra gain for the person because it adds something to their life, and adds something substantial if religion is important for them. [BA-M] offers an advantage for the person in that respect. (Turkey: GUD, BA-M therapist) “I would say using Behavioural Activation integrated with values seems good. This was a plus from my point of view.” (Turkey: ZM, BA-M therapist) “I felt it reaffirming like […] in some ways him [therapist] not being of the faith made that a bit easier because it made some of that like you know that inherent kind of judgement that […] might have had experience in the past. That wasn’t brought into the room. (UK: NH, NHS service user, female, completed) “it just changed my perspective on how well we can alter it when we need to. […] there’s no harm in incorporating faith-based activities and just anything that helps the client. You know, you do not want to feel like you are enforcing it on anyone or they are being judged. I think that was my own beliefs around it that came into it (UK: TI, NHS therapist, female) In Pakistan therapists and clients are mostly both from Muslim backgrounds, because of this shared understanding and trust it is easy for clients to stay engaged with therapy (Pakistan: TL, therapist, female)
***Increasing motivation and meaning***	“therapeutic values and like religious teachings are very similar in that like what we’re saying to do for the treatment for depression, Islam like sort of teaches you to do anyway and the religion sort of can help you in that way as well. So like bringing the two together was really helpful.” (UK: ST, NHS therapist, female) “It brought me back to my normal self and basically I was more motivated to going out and enjoying life, reading Quran and having friends, talking to my brothers and sisters.” (UK: NI, NHS service user, male, completed) “it helped me kind of realign to a meaning…gave me grounding of the strengths I had…there’s talk about like destiny and those elements that are very kind of strong in truth to my faith.” (UK: NH, NHS service user, female, completed)
***Negative feedback***	“I don’t think she had empathy […The therapist] could almost have asked that question and said, how did you feel about that and then maybe I could have answered it a bit more openly. […]I don’t want my religion to be forced on me […]. You know, it does help me, but obviously I’m still struggling.” (UK: BJ, NHS service user, male, dropped out) “they wanted to kind of like veer off [religion] and talk about other things so that was quite difficult at the beginning, but I got better at it. I think sometimes it made them feel uncomfortable in the sense that they were not prepared for it I do not think. (UK: BI, VSO therapist, female) “Doing activities created a routine but the voice that continued in my mind, there was no work on that” (Pakistan: HN, service user, female, completed) I felt like a student again—someone who had to be given homework: ‘Do this, do that.’ I mean, I really am not saying this in order to run it down. I felt a bit like, ‘Am I wasting my time?’ And of course [we must remember that] I was already in a state of depression, in a state of extreme despair, so I had thoughts like ‘How useful is this going to be?’” (Turkey: Client Zeynep, female, completed)
***Comparisons with previous*** ***experience***	“I feel like it was a lot more client-centred therapy” (UK: AC, NHS service user, female, completed) “the person I spoke to then was helpful but some part of it did not actually fit for me and with the religious part of it, it helped me to find peace inside of me. (UK: AT, female, VSO service user, completed) “I think it’s just more authentic and I think you relate on a more, you know, in more depth.” (UK: KT, VSO service user, female, completed) “I have seen practically change […] they were completely different, confused and now when they come to the centre, they are so happy. (UK: MH, VSO therapist, female) “I have seen long-term changes in clients. In Pakistan some individuals have a strong religious inclination, BA-M helps them connect with their spirituality in a way that other therapies I have used do not” (Pakistan, OI, PIMH therapist, female) “it puts more emphasis on these values, or why the person was left in that depressive cycle, and how they are going to get out of it. […] If I were to compare it with CBT, I would say this [BA-M] openly takes account [of values] and does so at a much earlier stage and in a more concrete fashion through the Behavioural Activation values scale.” (Turkey: UI, BA-M therapist) I was a bit worried because I do not identify myself as religious. With this, I would be introducing something I’m not adequately knowledgeable about into the therapy, and I was concerned about what I was going to do about it. In fact, this approach is not like that. Later, when I realised this, I felt more comfortable, and frankly, after applying it, the subject became clearer for me, and I became less concerned.” (Turkey: UI, BA-M therapist)

Data on a minority of negative experiences or views (Table 5) supported the need for fidelity to the BA-M protocol on client-centred engagement: two UK clients dropped out of therapy because their therapist made assumptions about their religious values rather than exploring these through a Values Assessment in line with the protocol. A small number of therapists and clients expressed doubts about the behavioural focus, which could seem prescriptive and not relevant to reducing unhelpful thoughts.

Qualitative data (Table 5) also revealed the mechanisms through which BA-M improved therapy outcomes, many of which overlapped with the reasons for improved engagement. Therapists pointed to the significance of religion as a core value to patients they had treated and how the approach could promote positive religious coping while challenging negative interpretations of religious teachings that contributed to depression, such as feelings of being punished. Engagement with religious identity could thus reveal contributory beliefs that might not be apparent to practitioners who did not recognise the impact of religious values. Therapists felt that having permission to recognise religion as a legitimate value and engage with this aspect of patients’ identity provided substantial benefits to service users and themselves. Patients pointed to the improved relationship with their therapists, compared to previous experiences. This was reflected in feedback from therapists, who felt they had changed their misconceptions about engaging with religion through using BA-M. For therapists in Pakistan, the shared religious background of therapists and clients was felt to contribute to increased engagement. An integrative summary of key findings from quantitative and qualitative analysis is provided in Supplementary Table G.

### Adverse events

No adverse events were reported by any site involved in the studies.

## Discussion

Our trial results show the greater effectiveness of BA-M compared to standard CBT and social interventions and confirm growing evidence that culturally adapted therapies are more effective than standard approaches (Anik et al., 2021). Effect sizes of PHQ-9 change scores are clinically significant at the population level (Löwe et al., 2004b) and, for most participants, at the individual level (Kounali et al., 2022). BA-M therapy reduced depression symptoms more than TAU by around 3 points on PHQ-9, and this appears to be because it motivates patients to continue engagement with an effective treatment.

Subgroup analyses to account for some variations across studies indicated that these had little impact on results. All studies included CBT as TAU; however, in the UK, TAU also included social interventions in community-based voluntary sector services. Our findings indicated minimal effect of variation due to TAU on the results. Therapist allocation to avoid contamination in the Turkey study might be considered to raise a potential risk of bias. A multilevel intercept model that accounted for therapist-level clustering, however, indicated that variance due to the therapists was low (Anik, 2022).

In the Pakistan trial, the difference between baseline activation scores in the treatment and control group could potentially have affected outcomes, as it is possible that the BA-M group was more inclined to be active. A study using BADS-SF in young people, however, found that changes in activation relative to one’s own baseline were clinically meaningful rather than between-person changes (Fisher et al., 2025).

The notable difference in retention rates in the Pakistan and UK trials may be related to contextual differences between the two samples and sites. In Pakistan, 72% of retained patients were classified as from higher socioeconomic backgrounds (Dawood et al., 2023), whereas available socioeconomic data for UK patients showed all were from deprived areas and, therefore, likely to have less capacity to engage with treatment. UK-wide data show that only a third of patients from deprived areas complete treatment for depression and anxiety (Baker and Kirk-Wade, 2024).

The Pakistan trial was also conducted at the beginning of the COVID-19 lockdown period (Dawood et al., 2023), during which limited mental healthcare services were available, possibly increasing client motivation to continue engagement. Furthermore, in terms of cultural context, all therapists in Pakistan shared the same religious background as patients, which can contribute to increased levels of trust (Mir et al., 2015). In the UK, there was more diversity among therapists and this may also have contributed to lower engagement; there is some evidence that culturally adapted treatments have more impact in contexts where the adaptation is for the majority population than for a minority group (Anik et al., 2021).

Qualitative findings indicate that BA-M can support mental healthcare services and practitioners to change their perspectives and recognise religious identity as a social factor that can influence mental health. The improved engagement described by patients and practitioners for BA-M appears to be a key mechanism for improved outcomes; our findings indicate the importance within this of therapist support for patients to develop “positive religious coping” (Pargament, 1997; Pargament et al., 2000) that provides meaning, resilience and hope. This engagement with patients’ religious identity also enables therapists to challenge “negative religious coping,” that is, feelings of despair or of being abandoned or punished, that can hinder positive religious coping (Pargament, 1997). These dynamics are unlikely to be apparent to therapists who do not engage with religious beliefs (Mir et al., 2019).

These findings on the mechanism of positive religious coping confirm that congruence between patient values and their meaning-making related to stressful events can help reduce depressive symptoms (Marco et al., 2021). BA-M outcomes also align with self-determination theory, in which better therapy outcomes are predicted when individuals experience their goals as freely chosen and personally meaningful (Zuroff et al., 2017).

The evidence from our trials, based on robust quantitative and qualitative methods, has important implications for global mental health in terms of practice and policy engagement with religious identity. This study provides multi-country randomised evidence that faith-sensitive, culturally adapted BA significantly improved depression outcomes and engagement among three diverse Muslim populations. Further research could usefully explore the effectiveness of BA-M across additional geographical contexts. The intervention was designed to be applicable across diverse Muslim sects (Mir et al., 2015); however, further research on its acceptability and effectiveness within specific Muslim sects would also be helpful.

Given the importance of religious identity to most people at the global level (Pew Research Centre, 2025), its recognition as a factor affecting mental health appears to be overdue. Attention to religion is also currently either absent or marginal within international mental health policy and guidance, which may conceptualise this determinant of health only in terms of potential partnerships between healthcare providers and religious organisations, rather than a factor influencing treatment interventions (World Health Organization, 2021, 2025).

Although “culturally appropriate care” is promoted by the World Health Organisation, the significance of patients’ religious identity as a resource for mental health is not specifically acknowledged within current guidance. BA-M therapy has the potential to supplement and enhance this predominantly secular view of mental healthcare, providing a practical framework through which recovery from depression can be supported for those who view religion as a significant aspect of their identity.

There is considerable current evidence of “monumental levels of lost human capabilities and avoidable suffering” caused by failure to address social determinants of mental health, and providers have been urged to “enthusiastically embrace” interventions that engage with these in order to “leave no one behind” (Patel et al., 2018). The recognition of Islam as a legitimate and valued identity within BA-M also goes some way to addressing the Islamophobia that is known to adversely influence Muslim mental health in the UK (Younis and Jadhav, 2020; The Lantern Initiative CIC et al., 2021) and other contexts in which Muslims are minorities (Rehman and Hanley, 2023). There is strong evidence that minority religious and ethnic populations experience cumulative exposure to racism that is recognised as an established determinant of mental health and is an additional barrier to recovery from depression (Wallace et al., 2016; Mir et al., 2024).

Through promoting the majority global population’s worldview on religion as an important value (Pew Research Center, 2025), BA-M may be seen to contribute to inclusive and antiracist practice in mental healthcare and research. In this respect, BA-M builds on existing conceptual frameworks for cultural adaptation that systematically modify interventions to ensure compatibility with the cultural patterns, meanings and values of specific client groups. These can include faith-sensitive approaches, but do not directly address the social context of epistemic power and privilege in terms of racism or the assumption that core elements and principles of existing interventions are universally applicable (Anik et al., 2021; Mishu et al., 2023). In contrast, “decolonial adaptations centre equity, contextual resonance and community self-determination.” Such adaptations value indigenous knowledge and promote epistemic justice, directly addressing the colonial legacies that inform racism (Agudelo-Hernández et al., 2025).

Muslims constitute the second-largest faith group globally (Pew Research Center, 2025). As a therapy that has been adapted to meet the specific needs of this population, BA-M responds to significant and widespread concerns about how health inequalities are created and maintained (Mir et al., 2024). It does this through decolonising knowledge about appropriate treatment for depression and transforming interactions between therapists and service users, as well as between healthcare providers and the Muslim populations they serve. Furthermore, our evidence that this approach can be delivered by non-specialist mental health practitioners in third sector organisations has important implications for widening the pool of therapy providers globally. As such, it addresses a key concern to increase the innovative use of trained non-specialist practitioners (Patel et al., 2018), who are also more likely to engage with the religious identity of service users than mainstream services (Mir and Sheikh, 2010).

### Limitations

The Pakistan trial demonstrated a significant imbalance in baseline activation scores between intervention and control groups, although depression levels were similar. This could have introduced a bias in relation to the intervention; further research could usefully evaluate BA-M in more closely matched groups in Pakistan. Variability in TAU between the UK and other studies and non-random therapist allocation to avoid contamination in the Turkey study may also be seen as potential sources of bias.

We were able to detect a treatment effect despite the small sample size in Turkey; however, as a feasibility trial, the study did not include a formal power calculation.

Due to a lack of resources, qualitative interviews and peer support sessions explored fidelity for BA-M but not for the TAU arm. This lack of monitoring in the TAU may have resulted in variability in how usual care was delivered, adversely affecting internal validity.

## Conclusion

Religious identity currently receives insufficient attention in global mental health policy and guidance. This study advances global mental health scholarship by providing multi-country randomised evidence that BA-M significantly improved depression outcomes and engagement among Muslim populations compared to CBT or social interventions. Key therapeutic mechanisms involve positive religious coping and anti-racist practice. Improved outcomes reduce health inequalities experienced by Muslim populations and promote changes to practice that involve decolonising current knowledge about depression treatment.

BA-M can be delivered by non-specialists, increasing the capacity of healthcare staff to deliver depression treatment. These findings indicate a need to scale up delivery of BA-M in Muslim majority and minority contexts and provide a model for faith-sensitive depression treatment in other faith groups.

## Supporting information

10.1017/gmh.2026.10245.sm001 Mir et al. supplementary material Mir et al. supplementary material

## Data Availability

Trial data are available for the Turkey trial (Anik, 2022), Pakistan trial (Dawood et al., 2023 and https://doi.org/10.1186/ISRCTN35418604) and UK trial (Mir et al., 2023 and https://www.isrctn.com/ISRCTN54894017).

## Supplementary material

The supplementary material for this article can be found at http://doi.org/10.1017/gmh.2026.10245.

## Long descriptions

### Table 1. Long description

The table is organized into four columns: Sites and arms, Intervention dose (number of sessions), Provider type, and Supervision and fidelity assessment.

* Turkey:

- B A - M: At least 6 sessions; Individual C B T-trained therapists; Clinical supervision and Peer support meetings.

- T A U: At least 6 sessions; Individual C B T-trained therapists; Clinical supervision.

* Pakistan:

- B A - M: 6 to 12 sessions; C B T-trained therapists in government-funded clinics; Clinical supervision.

- T A U: 8 sessions; C B T-trained therapists in government-funded clinics; Clinical supervision.

* U K:

- B A - M: N H S: 6 sessions, V S O: 6 to 12 sessions; N H S: C B T-trained therapists, V S O: Staff trained in B A; Clinical supervision, Peer support meetings, and Qualitative interviews.

- T A U: N H S: 6 sessions, V S O: open-ended; N H S: C B T-trained therapists, V S O: mental healthcare staff; Clinical supervision.

Navigate back to Table 1..

### Figure 1. Long description

The image consists of three vertical panels, each representing a C O N S O R T flow diagram.

Top Panel: Turkey feasibility trial.

- Recruited n equals 23.

- An arrow points right to Ineligible n equals 1 (Not Muslim).

- An arrow points down to Eligible n equals 22.

- The flow splits into two groups: Randomised to B A dash M and analysed n equals 14, and Randomised to T A U and analysed n equals 8.

Middle Panel: Pakistan randomised control trial.

- Recruited n equals 154.

- An arrow points right to Ineligible n equals 40 (Did not meet inclusion criteria n equals 32; Refused to participate n equals 8).

- An arrow points down to Eligible n equals 114.

- The flow splits into two groups: Randomised to B A dash M and analysed n equals 53, and Randomised to T A U and analysed n equals 50.

- An arrow from the T A U group points right to Insufficient data n equals 11 (4 B A dash M; 7 T A U).

Bottom Panel: U K randomised control trial.

- Recruited n equals 193.

- An arrow points right to Ineligible n equals 49 (P H Q 9 and G A D 7 thresholds not met).

- An arrow points down to Eligible n equals 144.

- The flow splits into two groups: Randomised to B A dash M and analysed n equals 81, and Randomised to T A U and analysed n equals 60.

- An arrow from the T A U group points right to Insufficient data n equals 3 (T A U).

Navigate back to Figure 1..

### Table 2. Long description

The table consists of four columns: Characteristic, B A minus M (n equals 148), T A U (n equals 118), and P test value.

* Study distribution (n, percentage):

- Pakistan: B A minus M is 53 (35.8); T A U is 50 (42.4); P equals 0.48.

- Turkey: B A minus M is 14 (9.5); T A U is 8 (6.8).

- U K: B A minus M is 81 (54.7); T A U is 60 (50.8).

* Sex (n, percentage):

- Female: B A minus M is 116 (78.4); T A U is 85 (77.3); P equals 0.95.

- Male: B A minus M is 32 (21.6); T A U is 25 (22.7).

* Age (mean (S D)):

- B A minus M is 34.17 (10.42); T A U is 37.43 (12.71); P equals 0.054.

* Baseline P H Q minus 9 score (mean (S D)):

- B A minus M is 19.12 (4.54); T A U is 19.71 (3.87); P equals 0.28.

Navigate back to Table 2..

### Figure 2. Long description

The graph consists of two side-by-side panels. The Y-axis is labeled P H Q score, ranging from 0 to over 20. The X-axis is labeled Session, ranging from 0 to 11.

Left Panel (B A M): Individual patient trajectories are shown as light gray lines with red dots. The data points are densely clustered between scores of 10 and 25 at session 0. A thick blue regression line with black circular markers shows a steady linear decrease from a P H Q score of approximately 19 at session 0 to approximately 1 at session 11. A gray shaded area around the blue line at the end of the timeline indicates the confidence interval.

Right Panel (T A U): Individual patient trajectories are shown as light gray lines with teal dots. Similar to the first panel, there is a high density of starting scores. The thick blue regression line with black circular markers shows a similar downward trajectory, starting at a P H Q score of 20 at session 0 and ending near 2 at session 11. The slope appears slightly steeper in the middle sessions compared to the B A M group.

Navigate back to Figure 2..

### Table 3. Long description

The table contains four columns: Term, Estimate, 95% C I (Confidence Interval), and p-value.

* Intercept: Estimate -0.96, 95% C I -4.03 to 2.11, p-value 0.538.

* Turkey: Estimate 3.63, 95% C I 1.23 to 6.02, p-value 0.003.

* U K: Estimate 10.40, 95% C I 9.13 to 11.66, p-value less than 0.001.

* Baseline P H Q 9: Estimate 0.38, 95% C I 0.24 to 0.52, p-value less than 0.001.

* B A M Therapy: Estimate -2.72, 95% C I -3.93 to -1.51, p-value less than 0.001.

Navigate back to Table 3..

### Table 4. Long description

The table consists of four columns: Term, I R R, 95 percent C I, and p-value.

* The Intercept row shows an I R R of 7.59, a 95 percent C I of 6.95 to 8.28, and a p-value less than 0.001.

* The Turkey row shows an I R R of 0.81, a 95 percent C I of 0.69 to 0.96, and a p-value of 0.015.

* The U K row shows an I R R of 0.43, a 95 percent C I of 0.39 to 0.48, and a p-value less than 0.001.

* The B A - M Therapy row shows an I R R of 1.30, a 95 percent C I of 1.18 to 1.44, and a p-value less than 0.001.

Navigate back to Table 4..

### Table 5. Long description

The table consists of seven thematic rows.

1. Significance of religion in therapy: Quotes from U K, Pakistan, and Turkey emphasize that religion is core to identity and hope. One participant from Turkey notes that not incorporating religious values led to the need for a psychologist.

2. Legitimacy of religious identity: U K managers and therapists discuss the need to acknowledge religion as a priority and the flexibility to talk about it openly.

3. Religious coping: Participants from Pakistan and the U K express that therapy should include Islamic materials. A U K service user references the Quranic principle of changing one’s own situation while trusting Allah. A Turkish therapist mentions using Socratic dialogue to resolve feelings of being punished for sins.

4. Valuing client beliefs: Therapists in Turkey and Pakistan note that shared Muslim backgrounds or integrating values adds substantial gain. A U K service user felt reaffirmed when a non-faith therapist avoided inherent judgment.

5. Increasing motivation and meaning: U K participants describe how Islamic teachings and therapeutic values align to help treat depression and provide grounding.

6. Negative feedback: Some U K service users felt religion was forced or made them uncomfortable. A Turkish client felt like a student being given homework and questioned the utility of the activities during deep despair.

7. Comparisons with previous experience: Participants describe B A-M (Behavioral Activation) as more authentic and client-centered than previous therapies. A Turkish therapist compares it to C B T, noting that B A-M takes values into account earlier and more concretely.

Navigate back to Table 5..
