# Supplementary material for: Treating depression in Muslim communities: Meta-analysis of three trials of culturally adapted behavioural activation for Muslims (BA-M)
Source: Glob Ment Health (Camb). 2026 Jun 11;13:e137. doi: 10.1017/gmh.2026.10245 (PMC13433330; doi:10.1017/gmh.2026.10245)
Supplement: Supplementary file 1 [file S2054425126102453sup001.docx]

## Supplementary Material – Tables A-G

|  |  | | TAU | | | BA-M | | |
| --- | --- | --- | --- | --- | --- | --- | --- | --- |
| Number of sessions | Overall | | Pakistan | Turkey | UK | Pakistan | Turkey | UK |
| n (%) | | 266 | 50 | 8 | 60 | 53 | 14 | 81 |
| 1 | | 42 (15.8) | 0 (0.0) | 0 (0.0) | 31 (51.7) | 0 (0.0) | 0 (0.0) | 11 (13.6) |
| 2 | | 22 (8.3) | 0 (0.0) | 1 (12.5) | 6 (10.0) | 0 (0.0) | 2 (14.3) | 13 (16.0) |
| 3 | | 14 (5.3) | 0 (0.0) | 0 (0.0) | 8 (13.3) | 0 (0.0) | 0 (0.0) | 6 (7.4) |
| 4 | | 11 (4.1) | 0 (0.0) | 0 (0.0) | 4 (6.7) | 0 (0.0) | 1 (7.1) | 6 (7.4) |
| 5 | | 16 (6.0) | 1 (2.0) | 1 (12.5) | 5 (8.3) | 0 (0.0) | 0 (0.0) | 8 (9.9) |
| 6 | | 17 (6.4) | 0 (0.0) | 1 (12.5) | 3 (5.0) | 0 (0.0) | 1 (7.1) | 12 (14.8) |
| 7 | | 37 (13.9) | 14 (28.0) | 0 (0.0) | 1 (1.7) | 9 (17.0) | 1 (7.1) | 12 (14.8) |
| 8 | | 39 (14.7) | 15 (30.0) | 1 (12.5) | 0 (0.0) | 15 (28.3) | 1 (7.1) | 7 (8.6) |
| 9 | | 28 (10.5) | 12 (24.0) | 0 (0.0) | 0 (0.0) | 11 (20.8) | 2 (14.3) | 2 (2.5) |
| 10 | | 23 (8.6) | 3 (6.0) | 1 (12.5.) | 2 (3.3) | 6 (11.3) | 3 (21.4) | 4 (4.9) |
| 11 | | 3 (1.1) | 2 (4.0) | 1 (12.5) | 0 (0.0) | 1 (1.9) | 0 (0.0) | 0 (0.0) |
| 12 or more | | 14 (5.3) | 3 (6.0) | 2 (25.0) | 0 (0.0) | 11 (20.8) | 3 (21.4) | 0 (0.0) |

Table A: Number of sessions attended by study and therapy group

|  | level | Overall | <5 point reduction | >= 5 point reduction |
| --- | --- | --- | --- | --- |
| n |  | 266 | 92 | 173 |
| Therapy (%) | TAU | 118 (44.4) | 49 (53.3) | 69 (39.9) |
|  | BA-M | 148 (55.6) | 43 (46.7) | 104 (60.1) |

Table B: Clients stratified by at least 5 point reduction on PHQ-9 scores (baseline and last PHQ-9)

|  | level | Overall | <20% reduction | >= 20% reduction |
| --- | --- | --- | --- | --- |
| n |  | 266 | 83 | 182 |
| Therapy (%) | TAU | 118 (44.4) | 45 (54.2) | 73 (40.1) |
|  | BA-M | 148 (55.6) | 38 (45.8) | 109 (59.9) |

Table C: Clients stratified by at least 20% reduction of PHQ-9 scores baseline and last PHQ-9)

Since the study conducted in Turkey was a feasibility study, we undertook a sensitivity test to examine whether excluding Turkey study made a difference to the findings. Table D shows findings from ANCOVA with results from the Turkey study excluded- final PHQ-9 scores are adjusted for study site, baseline PHQ-9 and therapy group. Participants receiving BA-M therapy had significantly lower post-treatment PHQ-9 scores compared to the TAU, that is 2.88 points reduction (β: -2.88, 95% CI [-4.13, -1.63], which is very similar to when the Turkey study was included, where there was 2.72 points reduction (β: -2.72, 95% CI [-3.93, -1.51]).

| **Term** | **Estimate** | **95% CI** | **p-value** |
| --- | --- | --- | --- |
| (Intercept) | -1.27 | -4.46, 1.92 | 0.435 |
| UK | 10.41 | 9.15, 11.67 | <0.001 |
| Baseline PHQ-9 | 0.40 | 0.25, 0.55 | <0.001 |
| BA-M therapy | -2.88 | -4.13, -1.63 | <0.001 |

Table D: ANCOVA results for UK and Pakistan studies only

Since there were differences in how TAU was defined between organisations that delivered therapy within the UK study, a sensitivity analysis was conducted for the UK study adjusting for the effect of organisation on therapy undertaken. Tables E and F show final PHQ-9 scores adjusted for baseline PHQ-9 and therapy group. Table F further includes adjustment for the organisation that delivered the therapy. Results show that adjusting for the organisation had only minimal impact on therapy effect.

| **Term** | **Estimate** | **95% CI** | **p- value** |
| --- | --- | --- | --- |
| (Intercept) | 4.35 | -0.70, 9.39 | 0.091 |
| Baseline PHQ-9 | 0.66 | 0.41, 0.90 | <0.001 |
| BA-M Therapy | -3.36 | -5.23, -1.49 | 0.001 |

Table E: ANCOVA results for UK study

| **Term** | **Estimate** | **95% CI** | **p- value** |
| --- | --- | --- | --- |
| (Intercept) | 3.19 | -2.17, 8.54 | 0.241 |
| Baseline PHQ-9 | 0.68 | 0.44, 0.93 | <0.001 |
| BA-M Therapy | -3.39 | -5.26, -1.53 | <0.001 |
| Sector VSO | 1.19 | -0.70, 3.08 | 0.214 |

Table F: ANCOVA results with the UK study with organisation

| **KEY QUANTITATIVE RESULTS** | **QUALITATIVE RESULTS – EXAMPLE QUOTES** | |
| --- | --- | --- |
| **Final mean depression score** | **Positive religious coping** | |
| BA-M = 9.6 (SD=7.16)  TAU = 11.95 (SD=7.15) | Positive religious coping | *“What I found was a lot of the women were coming across as like you’ve been punished […]. According to some of these women you are supposed to be angels all the time and it’s like that’s not possible because you are human and that’s how you’ve been created and that you do sin.”* (UK, VSO therapist) |
|  | Significance of religion | *“ I said to myself, ‘it is because I have not incorporated these things into my life that I am seeing to a psychologist today.”* (Turkey, Service User)  *“it was great, as we are Muslim and we should understand things with respect to our religion”*  (Pakistan, Service User) |
|  |  |  |
| **Retention of service users (mean number of sessions)** | **Inclusive practice** | |
| BA-M = 6.6  TAU = 5.3 | Legitimacy of religious identity | *“there’s absolutely a need for it and I think it can be for certain clients an absolute priority and can make all the difference when somebody is acknowledging.* (UK, NHS manager) |
|  | Valuing client beliefs | *“there is automatically an extra gain for the person because it adds something to their life, and adds something substantial if religion is important for them.* (Turkey, BA-M therapist)  *“it just changed my perspective […] there’s no harm in incorporating faith-based activities and just anything that helps the client. […] you don’t want to feel like you’re enforcing it on anyone or they’re being judged. I think that was my own beliefs around it that came into it”* (UK, NHS therapist) |
|  | Reasons for dropout | *“I don’t think she had empathy […The NHS therapist] could almost have asked that question and said, how did you feel about that and then maybe I could have answered it a bit more openly. […]I don’t want my religion to be forced on me […]. You know, it does help me, but obviously I’m still struggling”.*  (UK, Service User) |
|  | Comparison with previous experience of therapy | *"I feel like it was a lot more client-centred therapy”* (UK, Service User)  *“the person I spoke to then was helpful but some part of it didn’t actually fit for me and with the religious part of it, it helped me to find peace inside of me.”* (UK, Service User)  *“I think it’s just more authentic and I think you relate on a more, you know, in more depth."*  (UK, Service User) |

***Table G: Integrative summary of key findings***
