# Supplementary material for: Treating depression in Muslim communities: Meta-analysis of three trials of culturally adapted behavioural activation for Muslims (BA-M)
Source: Glob Ment Health (Camb). 2026 Jun 11;13:e137. doi: 10.1017/gmh.2026.10245 (PMC13433330; doi:10.1017/gmh.2026.10245)
Supplement: Peer review history

# Reviewer report: Review: Treating depression in Muslim communities: Meta-analysis of three trials of culturally adapted behavioural activation for Muslims (BA-M) — R0/PR2

**DOI:** 10.1017/gmh.2026.10245.pr2

**Authors:** Jamal Magantor

**Affiliations:** Bath Spa University Academic Centre RAK, United Arab Emirates

Thank you for the opportunity to review this important and timely manuscript. The study addresses a critical gap in global mental health by evaluating a culturally adapted intervention for Muslim communities across diverse contexts (Turkey, Pakistan, and the UK). The focus on Behavioural Activation for Muslims (BA-M), its delivery by non-specialists, and its relevance to equity, scalability, and acceptability represents a valuable contribution to the field.

The manuscript has strong potential for publication in Cambridge Prisms: Global Mental Health. However, several substantive issues require clarification and strengthening before the paper can be considered for acceptance. My comments below are intended to be constructive and to support the authors in enhancing the rigor, transparency, and interpretability of the work.

1. Clarification of comparators and heterogeneity across trials

The description of treatment-as-usual (TAU) varies considerably across sites (e.g., CBT in Pakistan and Turkey; NHS CBT versus voluntary-sector social interventions in the UK). Given that comparator heterogeneity directly affects interpretation of treatment effects, this issue should be addressed more explicitly. I recommend:

• Including a clear comparative table summarizing intervention dose, content, provider type, supervision, and fidelity across all sites and arms.

• Explicitly discussing the implications of TAU variability for pooled analyses.

• Where possible, conducting or reporting sensitivity or subgroup analyses (particularly within the UK sample) or clearly justifying why such analyses are not feasible.

2. Randomisation and risk of bias in the Turkey feasibility trial

The allocation deviations in the Turkey feasibility trial (e.g., therapist assignment due to training or translation involvement) raise concerns regarding selection bias and contamination. As this trial contributes to the pooled analysis, its methodological limitations should be more transparently handled. Please:

• Explicitly discuss the potential bias introduced by non-random therapist allocation.

• Clarify whether therapist-level clustering was accounted for in the analysis.

• Consider reporting sensitivity analyses excluding the Turkey trial, or clearly justify its inclusion despite these limitations.

3. Statistical modelling and analytic transparency

The analytic strategy requires further clarification, particularly the decision to adjust for number of sessions attended. As session attendance is likely influenced by treatment allocation and may function as a mediator rather than a confounder, this choice risks post-randomisation bias. I encourage the authors to:

• Clearly justify the inclusion of session count in the primary model.

• Present a primary intention-to-treat analysis without post-randomisation covariates, alongside secondary or exploratory analyses examining engagement/session attendance as a process variable.

• Provide additional detail on the multi-level modelling approach, handling of repeated measures, and treatment of missing data.

4. Interpretation and clinical significance of effects

The manuscript would benefit from clearer and more consistent presentation of effect sizes and their clinical relevance. Differences reported across sections (e.g., pooled PHQ-9 change versus adjusted model coefficients) should be reconciled. Please consider:

• Presenting effect sizes with confidence intervals in a consistent format.

• Discussing whether observed differences meet accepted thresholds for clinically meaningful change.

• Framing effectiveness claims with appropriate caution, particularly given cross-context heterogeneity.

5. Integration of qualitative and quantitative findings

The qualitative data are a major strength of the manuscript and provide important insight into mechanisms of engagement, acceptability, and perceived legitimacy. However, the mixed-methods integration could be strengthened. I suggest:

• More explicitly linking qualitative themes (e.g., religious identity, therapeutic legitimacy, motivation) to quantitative outcomes such as engagement or symptom change.

• Clarifying qualitative sample sizes and resolving minor inconsistencies in reporting.

• Including discussion of any divergent or negative cases, if available, to enhance analytic balance.

6. Framing of broader claims (equity, decolonisation, policy)

The discussion raises important points regarding secular norms, structural exclusion of religion in mental health care, and decolonisation of psychological interventions. These arguments are compelling, but some claims would benefit from tighter linkage to the empirical findings. I recommend:

• Clearly distinguishing data-driven conclusions from broader interpretive or policy implications.

• Defining key concepts (e.g., “decolonising mental health”) more explicitly within the context of this study.

• Moderating absolute language to ensure conclusions remain proportionate to the evidence presented.

7. Minor clarifications and reporting issues

• Ensure consistency in trial descriptions and terminology across sections.

• Justify differences in screening and outcome measures across sites.

• Briefly describe how adverse events were monitored and defined.

• Confirm that reporting aligns with relevant CONSORT extensions (cluster and feasibility trials).

• Review wording to avoid overgeneralisation or unqualified claims of superiority.

________________________________________

Overall assessment

This manuscript addresses an important and underexplored area in global mental health and has clear potential for impact. The required revisions are substantive but feasible and, if addressed, will significantly strengthen the methodological rigor, interpretive clarity, and contribution of the paper. I encourage the authors to engage fully with these points, as the study is well positioned to make a meaningful contribution once revised.

# Reviewer report: Review: Treating depression in Muslim communities: Meta-analysis of three trials of culturally adapted behavioural activation for Muslims (BA-M) — R0/PR3

**DOI:** 10.1017/gmh.2026.10245.pr3

**Authors:** Paulette Henry

**Affiliations:** University of Guyana, Guyana

Acceptance with minor revisions

This paper makes a significant contribution to the global mental health literature by addressing a critical gap in faith-sensitive interventions. Its first major strength lies in its strong clinical relevance: by centring one of the world’s largest faith-based communities, the study advances a decolonised therapeutic approach that moves beyond dominant Western secular paradigms. Second, the intervention demonstrates clear scalability. Evidence that non-specialist staff can effectively deliver BA-M in the UK context points to a promising, cost-effective model for expanding mental health service capacity in low-resource and workforce-constrained settings. Finally, the mixed methods design substantially enhances the paper’s contribution. By integrating a quantitative meta-analysis demonstrating a 2.35-unit reduction in PHQ-9 scores with qualitative process evaluations, the study offers a methodologically rigorous and contextually sensitive account of both intervention effectiveness, along with the mechanisms underpinning observed change.

Recommendations for Revisions

I have highlighted the specific areas where I believe that the authors should provide more clarity to strengthen the final manuscript.

1. The Treatment as Usual (TAU) varied significantly across sites and ranged from structured CBT in Pakistan and Turkey to open-ended social interventions in the UK.

Recommendation: Provide a more detailed sensitivity analysis or discussion on how comparing BA-M against such different control conditions might have influenced the meta-analysis results.

2. The Pakistan trial demonstrated a statistically significant imbalance in baseline activation scores between intervention and control groups (p = 0.001), despite comparable baseline depression severity. This imbalance may have inflated the observed intervention effects by providing the treatment group with a structural advantage in readiness for behavioural change. In addition, the Turkey study was designed as a small feasibility trial (n = 22) and did not include a formal power calculation, limiting its capacity to detect stable treatment effects.

Recommendation: The authors should explicitly address how both the baseline activation imbalance in the Pakistan trial and the limited statistical power of the Turkey study may have influenced the pooled effect size in the meta-analysis. A sensitivity analysis or stratified discussion distinguishing feasibility from fully powered trials would strengthen the robustness and interpretability of the findings.

3. A notable disparity was noted in treatment engagement across study sites, with participants in Pakistan attending a mean of 9.0 sessions compared to only 3.83 sessions in the UK. This divergence raises important questions about contextual, structural, and cultural factors which may have shaped retention.

Recommendation: The authors should draw on the qualitative data to explain why retention was substantially lower in the UK setting, even though BA-M continued to outperform treatment as usual. Attention to factors such as service delivery models, competing life demands, expectations of care, and the role of faith integration in different sociocultural contexts would strengthen the interpretation of the findings and enhance their relevance for implementation across diverse health systems.

4. In the UK arm of the study, a variation in training was observed, with non-specialist providers receiving five days of general BA training and an additional two days specific to BA-M. This contrasts with other trials in which the intervention was delivered by trained psychologists.

Recommendation: Although the literature supports the delivery of BA by non-specialist providers, WHO task-sharing guidance emphasises the importance of clearly articulated competency assessment, supervision, and fidelity-monitoring mechanisms. Given the training pathway used in the UK, greater detail on how provider competence and intervention fidelity were assessed and maintained would strengthen confidence in cross-site comparability and align the study more closely with WHO quality-assurance standards (World Health Organisation, 2025).

# Editor report: Recommendation: Treating depression in Muslim communities: Meta-analysis of three trials of culturally adapted behavioural activation for Muslims (BA-M) — R0/PR4

**DOI:** 10.1017/gmh.2026.10245.pr4

**Affiliations:** Psychology, UNISA, South Africa

Dear Prof. Mir,

Your manuscript, “Treating depression in Muslim communities: meta-analysis of three trials of culturally adapted Behavioural Activation for Muslims (BA-M)”, has now been assessed. You will see that, while the reviewers find your work of interest, they have raised points that need to be addressed by a Major revision.

We invite you to revise your paper, carefully addressing the comments from the reviewers and the editor. Please ensure the results are accurately reported, any overstated conclusions are rewritten, and the limitations of the work are fully explained. When your revision is ready, please submit the updated manuscript and a point-by-point response. This will help us move to a swift decision.

Handling Editor comments to the author: 

This manuscript makes a meaningful contribution by addressing an important and underexplored area in global mental health. Provide some greater detail on how provider competence and intervention fidelity were assessed and maintained. I encourage the authors to engage fully with the points raised by the reviewers. 

Ensure you also follow our authorship policies:

Reviewer Comments:

Reviewer 1

Thank you for the opportunity to review this important and timely manuscript. The study addresses a critical gap in global mental health by evaluating a culturally adapted intervention for Muslim communities across diverse contexts (Turkey, Pakistan, and the UK). The focus on Behavioural Activation for Muslims (BA-M), its delivery by non-specialists, and its relevance to equity, scalability, and acceptability represents a valuable contribution to the field.

The manuscript has strong potential for publication in Cambridge Prisms: Global Mental Health. However, several substantive issues require clarification and strengthening before the paper can be considered for acceptance. My comments below are intended to be constructive and to support the authors in enhancing the rigor, transparency, and interpretability of the work.

1. Clarification of comparators and heterogeneity across trials

The description of treatment-as-usual (TAU) varies considerably across sites (e.g., CBT in Pakistan and Turkey; NHS CBT versus voluntary-sector social interventions in the UK). Given that comparator heterogeneity directly affects interpretation of treatment effects, this issue should be addressed more explicitly. I recommend:

• Including a clear comparative table summarizing intervention dose, content, provider type, supervision, and fidelity across all sites and arms.

• Explicitly discussing the implications of TAU variability for pooled analyses.

• Where possible, conducting or reporting sensitivity or subgroup analyses (particularly within the UK sample) or clearly justifying why such analyses are not feasible.

2. Randomisation and risk of bias in the Turkey feasibility trial

The allocation deviations in the Turkey feasibility trial (e.g., therapist assignment due to training or translation involvement) raise concerns regarding selection bias and contamination. As this trial contributes to the pooled analysis, its methodological limitations should be more transparently handled. Please:

• Explicitly discuss the potential bias introduced by non-random therapist allocation.

• Clarify whether therapist-level clustering was accounted for in the analysis.

• Consider reporting sensitivity analyses excluding the Turkey trial, or clearly justify its inclusion despite these limitations.

3. Statistical modelling and analytic transparency

The analytic strategy requires further clarification, particularly the decision to adjust for number of sessions attended. As session attendance is likely influenced by treatment allocation and may function as a mediator rather than a confounder, this choice risks post-randomisation bias. I encourage the authors to:

• Clearly justify the inclusion of session count in the primary model.

• Present a primary intention-to-treat analysis without post-randomisation covariates, alongside secondary or exploratory analyses examining engagement/session attendance as a process variable.

• Provide additional detail on the multi-level modelling approach, handling of repeated measures, and treatment of missing data.

4. Interpretation and clinical significance of effects

The manuscript would benefit from clearer and more consistent presentation of effect sizes and their clinical relevance. Differences reported across sections (e.g., pooled PHQ-9 change versus adjusted model coefficients) should be reconciled. Please consider:

• Presenting effect sizes with confidence intervals in a consistent format.

• Discussing whether observed differences meet accepted thresholds for clinically meaningful change.

• Framing effectiveness claims with appropriate caution, particularly given cross-context heterogeneity.

5. Integration of qualitative and quantitative findings

The qualitative data are a major strength of the manuscript and provide important insight into mechanisms of engagement, acceptability, and perceived legitimacy. However, the mixed-methods integration could be strengthened. I suggest:

• More explicitly linking qualitative themes (e.g., religious identity, therapeutic legitimacy, motivation) to quantitative outcomes such as engagement or symptom change.

• Clarifying qualitative sample sizes and resolving minor inconsistencies in reporting.

• Including discussion of any divergent or negative cases, if available, to enhance analytic balance.

6. Framing of broader claims (equity, decolonisation, policy)

The discussion raises important points regarding secular norms, structural exclusion of religion in mental health care, and decolonisation of psychological interventions. These arguments are compelling, but some claims would benefit from tighter linkage to the empirical findings. I recommend:

• Clearly distinguishing data-driven conclusions from broader interpretive or policy implications.

• Defining key concepts (e.g., “decolonising mental health”) more explicitly within the context of this study.

• Moderating absolute language to ensure conclusions remain proportionate to the evidence presented.

7. Minor clarifications and reporting issues

• Ensure consistency in trial descriptions and terminology across sections.

• Justify differences in screening and outcome measures across sites.

• Briefly describe how adverse events were monitored and defined.

• Confirm that reporting aligns with relevant CONSORT extensions (cluster and feasibility trials).

• Review wording to avoid overgeneralisation or unqualified claims of superiority.

________________________________________

Overall assessment

This manuscript addresses an important and underexplored area in global mental health and has clear potential for impact. The required revisions are substantive but feasible and, if addressed, will significantly strengthen the methodological rigor, interpretive clarity, and contribution of the paper. I encourage the authors to engage fully with these points, as the study is well positioned to make a meaningful contribution once revised. 

Reviewer 2

Comments 

This paper makes a significant contribution to the global mental health literature by addressing a critical gap in faith-sensitive interventions. Its first major strength lies in its strong clinical relevance: by centring one of the world’s largest faith-based communities, the study advances a decolonised therapeutic approach that moves beyond dominant Western secular paradigms. Second, the intervention demonstrates clear scalability. Evidence that non-specialist staff can effectively deliver BA-M in the UK context points to a promising, cost-effective model for expanding mental health service capacity in low-resource and workforce-constrained settings. Finally, the mixed methods design substantially enhances the paper’s contribution. By integrating a quantitative meta-analysis demonstrating a 2.35-unit reduction in PHQ-9 scores with qualitative process evaluations, the study offers a methodologically rigorous and contextually sensitive account of both intervention effectiveness, along with the mechanisms underpinning observed change.

Recommendations for Revisions

I have highlighted the specific areas where I believe that the authors should provide more clarity to strengthen the final manuscript.

1. The Treatment as Usual (TAU) varied significantly across sites and ranged from structured CBT in Pakistan and Turkey to open-ended social interventions in the UK.

Recommendation: Provide a more detailed sensitivity analysis or discussion on how comparing BA-M against such different control conditions might have influenced the meta-analysis results.

2. The Pakistan trial demonstrated a statistically significant imbalance in baseline activation scores between intervention and control groups (p = 0.001), despite comparable baseline depression severity. This imbalance may have inflated the observed intervention effects by providing the treatment group with a structural advantage in readiness for behavioural change. In addition, the Turkey study was designed as a small feasibility trial (n = 22) and did not include a formal power calculation, limiting its capacity to detect stable treatment effects.

Recommendation: The authors should explicitly address how both the baseline activation imbalance in the Pakistan trial and the limited statistical power of the Turkey study may have influenced the pooled effect size in the meta-analysis. A sensitivity analysis or stratified discussion distinguishing feasibility from fully powered trials would strengthen the robustness and interpretability of the findings.

3. A notable disparity was noted in treatment engagement across study sites, with participants in Pakistan attending a mean of 9.0 sessions compared to only 3.83 sessions in the UK. This divergence raises important questions about contextual, structural, and cultural factors which may have shaped retention.

Recommendation: The authors should draw on the qualitative data to explain why retention was substantially lower in the UK setting, even though BA-M continued to outperform treatment as usual. Attention to factors such as service delivery models, competing life demands, expectations of care, and the role of faith integration in different sociocultural contexts would strengthen the interpretation of the findings and enhance their relevance for implementation across diverse health systems.

4. In the UK arm of the study, a variation in training was observed, with non-specialist providers receiving five days of general BA training and an additional two days specific to BA-M. This contrasts with other trials in which the intervention was delivered by trained psychologists.

Recommendation: Although the literature supports the delivery of BA by non-specialist providers, WHO task-sharing guidance emphasises the importance of clearly articulated competency assessment, supervision, and fidelity-monitoring mechanisms. Given the training pathway used in the UK, greater detail on how provider competence and intervention fidelity were assessed and maintained would strengthen confidence in cross-site comparability and align the study more closely with WHO quality-assurance standards (World Health Organisation, 2025). 

Kind regards

Dr Limkile Mpofu

Handling Editor

Cambridge Prisms: Global Mental Health

# Editor report: Decision: Treating depression in Muslim communities: Meta-analysis of three trials of culturally adapted behavioural activation for Muslims (BA-M) — R0/PR5

**DOI:** 10.1017/gmh.2026.10245.pr5

**Affiliations:** Johns Hopkins University Bloomberg School of Public Health, United States

No accompanying comment.

# Reviewer report: Review: Treating depression in Muslim communities: Meta-analysis of three trials of culturally adapted behavioural activation for Muslims (BA-M) — R1/PR7

**DOI:** 10.1017/gmh.2026.10245.pr7

**Authors:** Jamal Magantor

**Affiliations:** Bath Spa University Academic Centre RAK, United Arab Emirates

Peer Review Report

Manuscript Title: Treating depression in Muslim communities: meta-analysis of three trials of culturally adapted Behavioural Activation for Muslims (BA-M)

________________________________________

First, I would like to sincerely thank the Editor for the opportunity to review this highly important and impactful manuscript. I also commend the authors for addressing a critically underrepresented yet globally significant issue—the role of faith-sensitive interventions in treating depression among Muslim populations.

This manuscript represents a substantial and timely contribution to global mental health literature. The integration of randomized controlled trials across three distinct sociocultural contexts (UK, Pakistan, and Turkey), combined with patient-level meta-analysis and qualitative process evaluation, demonstrates a commendable level of methodological rigor and innovation. The development and evaluation of Behavioural Activation for Muslims (BA-M) is both conceptually compelling and practically relevant, particularly in advancing culturally responsive and decolonizing approaches to psychological treatment.

Below, I provide detailed and constructive feedback to further strengthen the manuscript.

________________________________________

Major Comments

1. Strengthen Conceptual Framing of “Religious Identity as Mechanism”

The manuscript compellingly argues that engagement with religious identity improves outcomes; however, the mechanism remains somewhat diffuse and largely inferred.

Suggestion:

• Explicitly articulate how religious identity functions as a therapeutic mechanism:

o Is it through meaning-making?

o Enhanced motivation and adherence?

o Cognitive restructuring via “positive religious coping”?

• Consider anchoring this mechanism within established frameworks such as:

• Pargament’s Religious Coping Theory

• Meaning-Making Models in Depression

• Self-Determination Theory (values congruence)

This will elevate the manuscript from descriptive effectiveness to mechanism-driven intervention science.

________________________________________

2. Clarify Meta-Analytic Approach and Statistical Rigor

The study is described as a “patient-level meta-analysis,” which is a major strength, but methodological clarity could be improved.

Concerns & Suggestions:

• Clarify whether this constitutes a true Individual Participant Data (IPD) meta-analysis versus pooled regression.

• Provide justification for:

• Not accounting for therapist-level clustering (especially given cluster randomization in Turkey)

• Expand on handling of:

• Missing baseline data (imputation strategy appears minimal)

• Clarify heterogeneity assessment across studies (currently underdeveloped)

Strengthening this section will significantly improve statistical credibility, especially for high-impact journals.

________________________________________

3. Address Potential Sources of Bias More Explicitly

While limitations are acknowledged, several sources of bias require deeper discussion:

• Non-blinded trials → risk of expectancy effects

• Variability in TAU (especially UK social interventions vs CBT)

• Baseline imbalance in Pakistan (activation scores)

• Therapist allocation in Turkey (non-random elements)

Suggestion:

Include a more explicit risk-of-bias reflection, possibly aligned with CONSORT or Cochrane domains.

________________________________________

4. Expand Theoretical Positioning within Global Mental Health

The manuscript makes strong claims about decolonizing mental health, which is commendable, but could be theoretically sharpened.

Suggestion:

• Clarify distinction between:

• Cultural adaptation

• Faith-sensitive therapy

• Decolonial practice

• Avoid overgeneralization—some claims (e.g., “challenging Western dominance”) may benefit from more balanced academic framing.

This will strengthen the manuscript’s positioning in critical global mental health discourse.

________________________________________

5. Strengthen Generalizability Claims

The manuscript implies global applicability of BA-M.

Concern:

• Samples are limited to:

• UK minority Muslim population

• Pakistan majority population

• Turkey feasibility sample (small)

Suggestion:

• Clarify boundaries of generalizability

• Acknowledge cultural heterogeneity within Muslim populations (e.g., sects, regional differences)

________________________________________

Methodological Suggestions

6. Clarify Intervention Fidelity Across Sites

While fidelity for BA-M is discussed, TAU fidelity is not equally monitored.

Suggestion:

• Acknowledge this imbalance more explicitly

• Discuss implications for internal validity

(Supplementary materials partially address this but should be integrated into the main text for clarity)

________________________________________

7. Expand on Qualitative Analysis Integration

The qualitative findings are rich but somewhat under-integrated.

Suggestion:

• Strengthen explicit linkage between qualitative themes and quantitative outcomes

• Consider a brief joint display or integrative summary of findings

This would enhance the mixed-methods rigor.

________________________________________

Results Section Suggestions

8. Improve Clarity of Statistical Reporting

Some statistical statements are difficult to interpret due to formatting issues.

Examples:

• ANCOVA reporting includes typographical inconsistencies

• Sentence structure around effect sizes is unclear

Suggestion:

• Standardize reporting (β, CI, p-values clearly separated)

• Ensure consistency across tables and narrative

________________________________________

9. Refine Presentation of Effect Sizes

The clinical significance is mentioned but could be strengthened.

Suggestion:

• Explicitly interpret effect sizes in clinical terms

• Compare with established CBT benchmarks

________________________________________

Discussion Strengthening

10. Sharpen Contribution Statement

The manuscript would benefit from a clearer, high-impact positioning.

Suggested sentence:

“This study advances global mental health scholarship by providing multi-country randomized evidence that faith-sensitive, culturally adapted behavioural activation significantly improves depression outcomes and engagement among Muslim populations.”

________________________________________

11. Balance Strength of Claims

Some statements are slightly overstated.

Suggestion:

• Moderate language around:

• “Transforming global mental health systems”

• “Decolonising knowledge”

This ensures credibility without diminishing impact.

________________________________________

Other Comments

• Correct typographical and formatting inconsistencies throughout (notably in Methods and Results)

• Ensure consistency in terminology (BA-M vs adapted BA)

• Remove duplicated or fragmented sentences in Discussion

• Standardize referencing format (some duplication of DOIs and links observed)

• Improve flow of long paragraphs in Discussion for readability

________________________________________

Final Recommendation

In conclusion, this manuscript is highly valuable, innovative, and publishable, addressing a critically important gap in culturally and faith-sensitive mental health interventions. The integration of multi-country RCT data, patient-level meta-analysis, and qualitative insights represents a significant contribution to both clinical psychology and global mental health.

The BA-M intervention is particularly noteworthy for its scalability, cultural relevance, and potential to reduce mental health inequities among Muslim populations worldwide.

However, several areas would benefit from refinement to further enhance the manuscript:

• Clearer articulation of therapeutic mechanisms (religious identity as a pathway)

• Strengthened methodological transparency (meta-analysis, clustering, bias)

• Refined theoretical positioning within global mental health discourse

• Improved clarity in statistical reporting and narrative flow

• More balanced and precise framing of broader claims

________________________________________

Editorial Decision: Minor to Moderate Revisions

I recommend acceptance pending minor to moderate revisions.

The manuscript is strong, timely, and impactful. With the suggested refinements, it has the potential to make a high-impact contribution to the field of culturally responsive and global mental health interventions.

I thank the authors for their excellent work and the Editor for the opportunity to review this important manuscript.

# Editor report: Recommendation: Treating depression in Muslim communities: Meta-analysis of three trials of culturally adapted behavioural activation for Muslims (BA-M) — R1/PR8

**DOI:** 10.1017/gmh.2026.10245.pr8

**Affiliations:** Psychology, UNISA, South Africa

Dear Prof. Mir,

Your manuscript, “Treating depression in Muslim communities: meta-analysis of three trials of culturally adapted Behavioural Activation for Muslims (BA-M)”, has now been assessed. You will see that, while the reviewers find your work of interest, they have raised points that require a Minor revision.

We invite you to revise your paper, carefully addressing the reviewers' and the editor’s comments. When your revision is ready, please submit the updated manuscript and a point-by-point response. This will help us move to a swift decision.

Reviewer Comments:

Reviewer 1

________________________________________

First, I would like to sincerely thank the Editor for the opportunity to review this highly important and impactful manuscript. I also commend the authors for addressing a critically underrepresented yet globally significant issue—the role of faith-sensitive interventions in treating depression among Muslim populations.

This manuscript represents a substantial and timely contribution to global mental health literature. The integration of randomized controlled trials across three distinct sociocultural contexts (UK, Pakistan, and Turkey), combined with patient-level meta-analysis and qualitative process evaluation, demonstrates a commendable level of methodological rigor and innovation. The development and evaluation of Behavioural Activation for Muslims (BA-M) is both conceptually compelling and practically relevant, particularly in advancing culturally responsive and decolonizing approaches to psychological treatment.

Below, I provide detailed and constructive feedback to further strengthen the manuscript.

________________________________________

Major Comments

1. Strengthen Conceptual Framing of “Religious Identity as Mechanism”

The manuscript compellingly argues that engagement with religious identity improves outcomes; however, the mechanism remains somewhat diffuse and largely inferred.

Suggestion:

• Explicitly articulate how religious identity functions as a therapeutic mechanism:

o Is it through meaning-making?

o Enhanced motivation and adherence?

o Cognitive restructuring via “positive religious coping”?

• Consider anchoring this mechanism within established frameworks such as:

• Pargament’s Religious Coping Theory

• Meaning-Making Models in Depression

• Self-Determination Theory (values congruence)

This will elevate the manuscript from descriptive effectiveness to mechanism-driven intervention science.

________________________________________

2. Clarify Meta-Analytic Approach and Statistical Rigor

The study is described as a “patient-level meta-analysis,” which is a major strength, but methodological clarity could be improved.

Concerns & Suggestions:

• Clarify whether this constitutes a true Individual Participant Data (IPD) meta-analysis versus pooled regression.

• Provide justification for:

• Not accounting for therapist-level clustering (especially given cluster randomization in Turkey)

• Expand on handling of:

• Missing baseline data (imputation strategy appears minimal)

• Clarify heterogeneity assessment across studies (currently underdeveloped)

Strengthening this section will significantly improve statistical credibility, especially for high-impact journals.

________________________________________

3. Address Potential Sources of Bias More Explicitly

While limitations are acknowledged, several sources of bias require deeper discussion:

• Non-blinded trials → risk of expectancy effects

• Variability in TAU (especially UK social interventions vs CBT)

• Baseline imbalance in Pakistan (activation scores)

• Therapist allocation in Turkey (non-random elements)

Suggestion:

Include a more explicit risk-of-bias reflection, possibly aligned with CONSORT or Cochrane domains.

________________________________________

4. Expand Theoretical Positioning within Global Mental Health

The manuscript makes strong claims about decolonizing mental health, which is commendable, but could be theoretically sharpened.

Suggestion:

• Clarify distinction between:

• Cultural adaptation

• Faith-sensitive therapy

• Decolonial practice

• Avoid overgeneralization—some claims (e.g., “challenging Western dominance”) may benefit from more balanced academic framing.

This will strengthen the manuscript’s positioning in critical global mental health discourse.

________________________________________

5. Strengthen Generalizability Claims

The manuscript implies global applicability of BA-M.

Concern:

• Samples are limited to:

• UK minority Muslim population

• Pakistan majority population

• Turkey feasibility sample (small)

Suggestion:

• Clarify boundaries of generalizability

• Acknowledge cultural heterogeneity within Muslim populations (e.g., sects, regional differences)

________________________________________

Methodological Suggestions

6. Clarify Intervention Fidelity Across Sites

While fidelity for BA-M is discussed, TAU fidelity is not equally monitored.

Suggestion:

• Acknowledge this imbalance more explicitly

• Discuss implications for internal validity

(Supplementary materials partially address this but should be integrated into the main text for clarity)

________________________________________

7. Expand on Qualitative Analysis Integration

The qualitative findings are rich but somewhat under-integrated.

Suggestion:

• Strengthen explicit linkage between qualitative themes and quantitative outcomes

• Consider a brief joint display or integrative summary of findings

This would enhance the mixed-methods rigor.

________________________________________

Results Section Suggestions

8. Improve Clarity of Statistical Reporting

Some statistical statements are difficult to interpret due to formatting issues.

Examples:

• ANCOVA reporting includes typographical inconsistencies

• Sentence structure around effect sizes is unclear

Suggestion:

• Standardize reporting (β, CI, p-values clearly separated)

• Ensure consistency across tables and narrative

________________________________________

9. Refine Presentation of Effect Sizes

The clinical significance is mentioned but could be strengthened.

Suggestion:

• Explicitly interpret effect sizes in clinical terms

• Compare with established CBT benchmarks

________________________________________

Discussion Strengthening

10. Sharpen Contribution Statement

The manuscript would benefit from a clearer, high-impact positioning.

Suggested sentence:

“This study advances global mental health scholarship by providing multi-country randomized evidence that faith-sensitive, culturally adapted behavioural activation significantly improves depression outcomes and engagement among Muslim populations.”

________________________________________

11. Balance Strength of Claims

Some statements are slightly overstated.

Suggestion:

• Moderate language around:

• “Transforming global mental health systems”

• “Decolonising knowledge”

This ensures credibility without diminishing impact.

________________________________________

Other Comments

• Correct typographical and formatting inconsistencies throughout (notably in Methods and Results)

• Ensure consistency in terminology (BA-M vs adapted BA)

• Remove duplicated or fragmented sentences in Discussion

• Standardize referencing format (some duplication of DOIs and links observed)

• Improve flow of long paragraphs in Discussion for readability

________________________________________

Final Recommendation

In conclusion, this manuscript is highly valuable, innovative, and publishable, addressing a critically important gap in culturally and faith-sensitive mental health interventions. The integration of multi-country RCT data, patient-level meta-analysis, and qualitative insights represents a significant contribution to both clinical psychology and global mental health.

The BA-M intervention is particularly noteworthy for its scalability, cultural relevance, and potential to reduce mental health inequities among Muslim populations worldwide.

However, several areas would benefit from refinement to further enhance the manuscript:

• Clearer articulation of therapeutic mechanisms (religious identity as a pathway)

• Strengthened methodological transparency (meta-analysis, clustering, bias)

• Refined theoretical positioning within global mental health discourse

• Improved clarity in statistical reporting and narrative flow

• More balanced and precise framing of broader claims

________________________________________

Editorial Decision: Minor to Moderate Revisions needed

The manuscript is strong, timely, and impactful. With the suggested refinements, it has the potential to make a high-impact contribution to the field of culturally responsive and global mental health interventions.

Thank you

Dr Limkile Mpofu

Handling Editor (Cambridge Prisms: Global Mental Health)

# Editor report: Decision: Treating depression in Muslim communities: Meta-analysis of three trials of culturally adapted behavioural activation for Muslims (BA-M) — R1/PR9

**DOI:** 10.1017/gmh.2026.10245.pr9

**Affiliations:** Johns Hopkins University Bloomberg School of Public Health, United States

No accompanying comment.

# Editor report: Recommendation: Treating depression in Muslim communities: Meta-analysis of three trials of culturally adapted behavioural activation for Muslims (BA-M) — R2/PR11

**DOI:** 10.1017/gmh.2026.10245.pr11

**Affiliations:** Psychology, UNISA, South Africa

Dear Prof. Mir,

We are pleased to confirm that your manuscript ID: GMH-2025-0431, Manuscript Title: “Treating depression in Muslim communities: meta-analysis of three trials of culturally adapted Behavioural Activation for Muslims (BA-M)”, has received an acceptance

decision from our Editorial Team. Congratulations on this significant

achievement! We truly appreciate your hard work and dedication in ensuring

that your research is scientifically sound and of high quality.

We will work closely with the corresponding author (Prof. Mir),

to complete the final steps toward publication. 

Sincerely,

Dr. Limkile Mpofu

Cambridge Prisms: Global Mental Health

# Editor report: Decision: Treating depression in Muslim communities: Meta-analysis of three trials of culturally adapted behavioural activation for Muslims (BA-M) — R2/PR12

**DOI:** 10.1017/gmh.2026.10245.pr12

**Affiliations:** Johns Hopkins University Bloomberg School of Public Health, United States

No accompanying comment.
